# A Comparative Evaluation of the Therapeutic Effects of Adenosine Triphosphate, Coenzyme Q10, Pyridoxine, and Thiamine Pyrophosphate in a Linezolid-Induced Peripheral Neuropathic Pain Model in Rats

**DOI:** 10.3390/ph19020341

**Published:** 2026-02-22

**Authors:** Habip Burak Ozgodek, Ramazan Ince, Agah Abdullah Kahramanlar, Bulent Yavuzer, Esra Tuba Sezgin, Renad Mammadov, Nuri Bakan, Halis Suleyman

**Affiliations:** 1 Department of Anesthesiology and Reanimation, Erzurum City Hospital, Erzurum 25070, Turkey; brk.ozgodek@gmail.com (H.B.O.); rmzn.ince@hotmail.com (R.I.); ultradr1@hotmail.com (A.A.K.); 2 Department of Pharmacology, Faculty of Medicine, Erzincan Binali Yıldırım University, Erzincan 24100, Turkey; bulent.yavuzer@erzincan.edu.tr (B.Y.); rmammadov@erzincan.edu.tr (R.M.); 3 Anesthesia Program, Vocational School of Health Services, Erzincan Binali Yıldırım University, Erzincan 24036, Turkey; esra.demir@erzincan.edu.tr; 4 Department of Medical Biochemistry, Faculty of Medicine, Erzincan Binali Yıldırım University, Erzincan 24100, Turkey; nuri.bakan@erzincan.edu.tr

**Keywords:** adenosine triphosphate, coenzyme Q10, linezolid, paw withdrawal threshold, pyridoxine, sciatic nerve, thiamine pyrophosphate

## Abstract

**Background/Objectives:** Linezolid is an oxazolidinone antibiotic whose prolonged use is associated with peripheral neuropathy, hyperlactatemia, and metabolic acidosis. These adverse effects are primarily linked to the inhibition of mitochondrial protein synthesis, respiratory chain dysfunction, and oxidative stress. Given the central role of impaired energy metabolism and redox imbalance in drug-induced peripheral neuropathy, therapeutic strategies targeting mitochondrial function are of particular interest. Accordingly, this study aimed to comparatively evaluate the effects of adenosine triphosphate (ATP), coenzyme Q10 (CoQ10), pyridoxine, and thiamine pyrophosphate (TPP) on linezolid-induced peripheral neuropathic pain in rats. **Methods:** Sixty male albino Wistar rats were assigned to ten groups: healthy (HG); ATP-only (ATPG, 5 mg/kg, intraperitoneally); CoQ10-only (CQ10G, 10 mg/kg, orally); pyridoxine-only (PDXG, 50 mg/kg, orally); TPP-only (TPPG, 20 mg/kg, intraperitoneally); linezolid-only (LZDG, 125 mg/kg, orally); linezolid+ATP (ATLG); linezolid+CoQ10 (CQLG); linezolid+pyridoxine (PXLG); and linezolid+TPP (TPLG). Treatments were administered once daily for ATP, CoQ10, and TPP, and twice daily for linezolid and pyridoxine for 14 days. Oxidative stress indices (MDA, tGSH, SOD, CAT) were quantified in the sciatic nerve using ELISA. Serum lactate dehydrogenase (LDH) activity and blood lactate levels were determined to evaluate metabolic disturbances. Mechanical paw withdrawal thresholds were measured using the Randall–Selitto test both before and after treatment. **Results:** Linezolid significantly reduced paw withdrawal thresholds and induced oxidative stress, antioxidant depletion, increased LDH activity, and hyperlactatemia. Co-treatment with ATP and CoQ10 attenuated oxidative stress but did not significantly improve linezolid-induced reductions in nociceptive thresholds. In contrast, pyridoxine partially alleviated linezolid-induced neuropathic pain and improved biochemical parameters. Notably, TPP exerted the most robust protective effect, preserving nociceptive thresholds and effectively normalizing oxidative stress and metabolic indices. **Conclusions:** These findings identify TPP as a promising therapeutic strategy for mitigating linezolid-induced peripheral neuropathic pain by targeting mitochondrial energy metabolism and pyruvate–lactate homeostasis.

## 1. Introduction

Linezolid, a synthetic antibiotic belonging to the oxazolidinone class, is indicated for the treatment of complicated and uncomplicated skin and soft tissue infections, community-acquired and hospital-acquired pneumonia, and drug-resistant Gram-positive infections [1]. Its antimicrobial activity is mediated by inhibition of protein synthesis through binding to the peptidyl transferase center of the 50S ribosomal subunit [2]. In addition to its antibacterial activity, linezolid inhibits mitochondrial protein synthesis [3]. Through inhibition of mitochondrial protein synthesis, it leads to a reduction in the activity of respiratory chain complexes [4]. Mitochondrial dysfunction in Schwann cells can induce adverse effects, including neuronal loss and degeneration of the myelin sheath [5,6,7]. Linezolid is also associated with adverse effects such as hyperlactatemia, peripheral neuropathy, and metabolic acidosis [3]. In case reports, Protti et al. reported linezolid-associated lactic acidosis accompanied by reduced global oxygen consumption and decreased activities of respiratory chain complexes I, III, and IV [8]. Santini et al. defined linezolid-associated lactic acidosis as an adverse effect resulting from lactate accumulation via anaerobic pathways due to inhibition of mitochondrial protein synthesis [9].

Peripheral neuropathy, one of the adverse effects associated with linezolid, is a common neurological disorder and represents a broad term encompassing any dysfunction of the peripheral nervous system [10]. When peripheral neuropathy is triggered by pharmacological agents, it is referred to as drug-induced peripheral neuropathy [11]. The pathogenesis of this type of neuropathy is primarily driven by oxidative stress and mitochondrial dysfunction [12]. These pathophysiological mechanisms are consistent with the proposed molecular basis of linezolid-induced neurotoxicity. Bressler et al. reported in clinical studies that prolonged use of linezolid is associated with severe peripheral and optic neuropathies [13]. Moreover, an association between lactic acidosis and peripheral neuropathies has been reported in the literature [14].

Adenosine triphosphate (ATP), which will be investigated for its effects on linezolid-induced peripheral neuropathic pain in rats, is synthesized in mitochondria through oxidative phosphorylation. The literature also emphasizes that mitochondrial respiratory function and ATP synthesis are potential targets that should be considered for the treatment of peripheral neuropathy [15].

The antioxidant effect of coenzyme Q10 (CoQ10), whose effects against linezolid-induced peripheral neuropathic pain will be investigated in rats in this study, arises from its ability to protect cellular organelles against oxidative damage via its ubiquinol form. The oxidized form of CoQ10 (ubiquinone) and its semi-reduced form (ubisemiquinone) contribute to the maintenance of cellular redox homeostasis [16]. Coenzyme Q10 (CoQ10) shows promise as a neuroprotective agent with the potential to enhance therapeutic efficacy in nervous system disorders by reducing oxidative stress and inflammation [17]. It facilitates electron transport during ATP synthesis and suppresses the production of reactive oxygen species (ROS). It has been reported to enhance the myelination of peripheral nerve fibers and to support peripheral nerve regeneration [18]. The literature indicates that antioxidant and anti-inflammatory supplementation, such as CoQ10, has been shown to reduce pain in patients with diabetic peripheral neuropathic pain [19].

Pyridoxine (vitamin B6), evaluated in the present study for its potential modulatory effects against linezolid-induced peripheral neuropathic pain in rats, is a water-soluble vitamin distributed across numerous dietary sources. It occurs in the form of pyridoxine, pyridoxal, and pyridoxamine [20]. Pyridoxine plays a critical role in axonal transport and neurotransmitter biosynthesis, and its deficiency has been consistently associated with the development of peripheral neuropathy [21]. Koker et al. clinically demonstrated that pyridoxine and pyridostigmine exert therapeutic benefits in the management of drug-induced peripheral neuropathy [22]. Déry et al. reported, in their case reports, that decreased pyridoxine levels may be associated with an elevated risk of neuropathy in patients [23].

Thiamine pyrophosphate (TPP), evaluated in the present study for its potential effects against linezolid-induced peripheral neuropathy and neuropathic pain in rats, represents the biologically active metabolite of thiamine (vitamin B1) [24]. Thiamine undergoes enzymatic conversion to its active form, TPP, via thiamine pyrophosphokinase within the body [25]. Accumulating evidence from previous studies indicates that TPP exhibits pronounced antioxidant and anti-inflammatory properties, thereby conferring protection to sciatic nerve tissue from injury mediated by oxidative stress and proinflammatory cytokines [26]. Collectively, these findings derived from the literature suggest that ATP, CoQ10, pyridoxine, and TPP may exert beneficial effects against linezolid-induced peripheral neuropathic pain. Moreover, a comprehensive review of the available literature revealed a lack of studies investigating the effects of these molecules on linezolid-associated neuropathy and neuropathic pain. The present study aims to investigate and comparatively evaluate the effects of ATP, CoQ10, pyridoxine, and TPP on linezolid-induced neuropathy-associated pain in rats.

## 2. Results

### 2.1. Mechanical Paw Withdrawal Threshold Results

Baseline mechanical paw withdrawal thresholds were comparable between all experimental groups, with no statistically significant differences detected prior to treatment. Following treatment, the linezolid-only group exhibited a pronounced reduction in paw withdrawal thresholds compared with the healthy control and all agent-only groups. Co-treatment with pyridoxine or TPP significantly attenuated the linezolid-induced decrease in paw withdrawal thresholds, as reflected by higher post-treatment values and smaller Δ (post–pre) reductions relative to the LZDG. In contrast, co-administration of ATP or CoQ10 did not result in a significant improvement in paw withdrawal thresholds when compared with the linezolid-only group.

**Figure 1:**
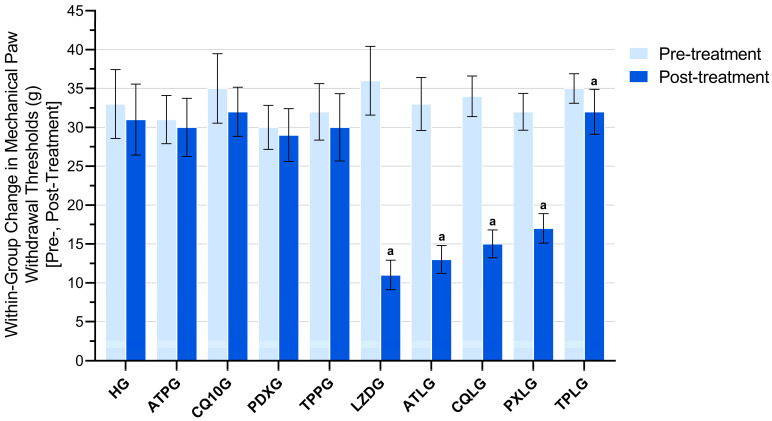
Comparison of pre- and post-treatment mechanical paw withdrawal thresholds within each group. Footnotes: All data are presented as mean ± SD (standard deviation). Within-group comparisons were performed using paired-samples *t*-tests. a indicates *p* < 0.05 post-treatment vs. pre-treatment. For all groups, *n* = 6. Abbreviations: HG, healthy group; ATPG, ATP-only group; CQ10G, coenzyme Q10-only group; PDXG, pyridoxine-only group; TPPG, TPP-only group; LZDG, linezolid-only group; ATLG, ATP + linezolid; CQLG, coenzyme Q10 + linezolid; PXLG, pyridoxine + linezolid; TPLG, TPP + linezolid; ATP, adenosine triphosphate; TPP, thiamine pyrophosphate.

Within-group analyses demonstrated that the linezolid-only group exhibited the greatest reduction in mechanical paw withdrawal thresholds, reflected by a pronounced negative change between pre- and post-treatment measurements (Δ = −25.00 ± 2.76 g; t = −22.213; *p* < 0.001; Cohen’s dz = −9.068). Significant within-group decreases were also observed in all linezolid co-treatment groups, including ATP + linezolid (ATLG; Δ = −20.00 ± 2.28 g; t = −21.483; *p* < 0.001; dz = −8.771), CoQ10 + linezolid (CQLG; Δ = −19.00 ± 4.15 g; t = −11.222; *p* < 0.001; dz = −4.581), and pyridoxine + linezolid (PXLG; Δ = −15.00 ± 1.41 g; t = −25.981; *p* < 0.001; dz = −10.607). In contrast, the TPP + linezolid group (TPLG) exhibited a significantly smaller reduction in paw withdrawal thresholds (Δ = −3.00 ± 1.79 g; t = −4.108; *p* = 0.009; dz = −1.677). No statistically significant pre–post-treatment differences were detected in the healthy control group (HG; Δ = −2.00 ± 6.99 g; *p* = 0.514) or in the agent-only groups, including ATPG (Δ = −1.00 ± 5.25 g; *p* = 0.661), CQ10G (Δ = −3.00 ± 4.86 g; *p* = 0.191), PDXG (Δ = −1.00 ± 2.61 g; *p* = 0.391), and TPPG (Δ = −2.00 ± 6.32 g; *p* = 0.474). These findings are summarized in Figure 1.

**Figure 2:**
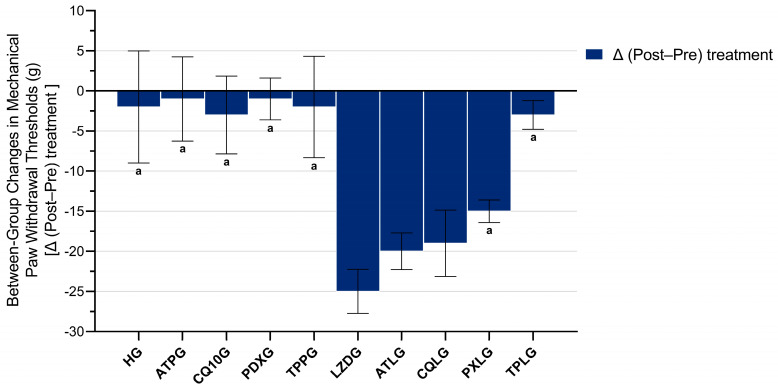
Between-group comparison of treatment-related changes in mechanical paw withdrawal thresholds (Δ = post–pre). Footnotes: All data are presented as mean ± SD (standard deviation). Between-group comparisons were conducted using Welch’s ANOVA followed by the Games–Howell post hoc test. a indicates *p* < 0.05 vs. LZDG. For all groups, *n* = 6. Abbreviations: HG, healthy group; ATPG, ATP-only group; CQ10G, coenzyme Q10-only group; PDXG, pyridoxine-only group; TPPG, TPP-only group; LZDG, linezolid-only group; ATLG, ATP + linezolid; CQLG, coenzyme Q10 + linezolid; PXLG, pyridoxine + linezolid; TPLG, TPP + linezolid; ATP, adenosine triphosphate; TPP, thiamine pyrophosphate; Δ, difference between post-treatment and pre-treatment values.

Between-group comparisons of Δ (post–pre) mechanical paw withdrawal thresholds demonstrated a significant overall treatment effect (Welch’s ANOVA, F(9, 20.12) = 51.216, *p* < 0.001). Post hoc Games–Howell analyses revealed that the linezolid-only group exhibited significantly greater reductions in paw withdrawal thresholds than the healthy control group (HG, *p* = 0.003) and all agent-only groups, including ATPG (*p* < 0.001), CQ10G (*p* < 0.001), PDXG (*p* < 0.001), and TPPG (*p* = 0.002). Relative to the LZDG, co-treatment with pyridoxine or TPP resulted in significantly smaller Δ reductions (PXLG vs. LZDG, *p* = 0.001; TPLG vs. LZDG, *p* < 0.001), whereas no significant differences in Δ values were observed between the LZDG and either the ATP + linezolid or the CoQ10 + linezolid groups (ATLG vs. LZDG, *p* = 0.110; CQLG vs. LZDG, *p* = 0.220). Further pairwise comparisons indicated that the Δ values in the TPP + linezolid group were comparable to those of the healthy control group (TPLG vs. HG, *p* = 1.000), whereas the Δ values observed in the pyridoxine + linezolid group were not significantly different from those of the healthy control group (PXLG vs. HG, *p* = 0.066). These results are presented in Figure 2.

### 2.2. Effects of ATP, CoQ10, Pyridoxine, and TPP on Relative Restoration of Mechanical Threshold

Under basal conditions, the HG and all single-agent treatment groups exhibited mechanical thresholds that were comparable to one another and close to physiological baseline values, indicating that administration of ATP, CoQ10, pyridoxine, or TPP alone did not significantly modify mechanical sensitivity in the absence of linezolid.

Following repeated administration of linezolid at a dose of 125 mg/kg twice daily, co-treatment strategies resulted in variable levels of relative restoration of the mechanical threshold. Co-treatment with ATP and CoQ10 was associated with low relative restoration (20% and 24%, respectively). Pyridoxine co-treatment produced a moderate level of relative restoration (40%). In contrast, TPP co-treatment maintained a high level of relative restoration (88%), comparable with basal conditions.

### 2.3. Biochemical Findings

#### 2.3.1. Analysis of MDA Levels in Sciatic Nerve Tissue

**Figure 3:**
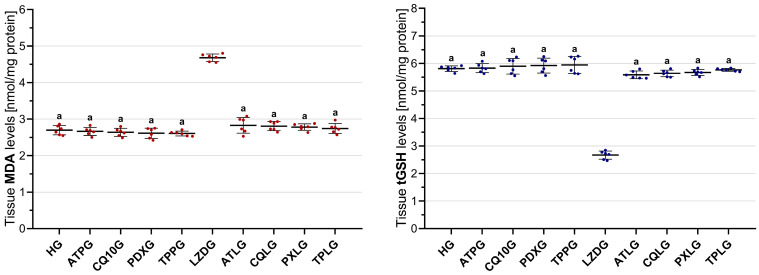
Effects of ATP, coenzyme Q10, pyridoxine, TPP, and linezolid on MDA and tGSH levels in rat sciatic nerve tissue. Footnotes: All values are expressed as mean ± SD (standard deviation). All statistical analyses were performed using Welch’s ANOVA followed by Games–Howell post hoc tests. a indicates *p* < 0.001 vs. LZDG. For all groups, *n* = 6. Abbreviations: HG, healthy group; ATPG, ATP-only group; CQ10G, coenzyme Q10-only group; PDXG, pyridoxine-only group; TPPG, TPP-only group; LZDG, linezolid-only group; ATLG, ATP + linezolid; CQLG, coenzyme Q10 + linezolid; PXLG, pyridoxine + linezolid; TPLG, TPP + linezolid; ATP, adenosine triphosphate; TPP, thiamine pyrophosphate; MDA, malondialdehyde; tGSH, total glutathione.

As illustrated in Figure 3, sciatic nerve MDA levels were highest in the linezolid-only group (LZDG, 4.68 ± 0.10). In contrast, significantly lower MDA levels were observed in the healthy control group (HG, 2.70 ± 0.13) and in all groups treated with individual agents, including ATPG (2.66 ± 0.11), CQ10G (2.64 ± 0.11), PDXG (2.61 ± 0.14), and TPPG (2.67 ± 0.07), with all comparisons versus the LZDG reaching statistical significance (*p* < 0.001).

Co-treatment with ATP (ATLG, 2.83 ± 0.22), CoQ10 (CQLG, 2.81 ± 0.13), pyridoxine (PXLG, 2.78 ± 0.09), and TPP (TPLG, 2.74 ± 0.14) significantly suppressed the linezolid-induced increase in MDA levels in sciatic nerve tissue when compared with the LZDG (all *p* < 0.001). However, no significant differences in MDA levels were found between the four co-treatment groups (all *p* > 0.05).

#### 2.3.2. Analysis of tGSH Levels in Sciatic Nerve Tissue

As depicted in Figure 3, sciatic nerve tGSH levels were significantly diminished in the linezolid-only group (LZDG, 2.67 ± 0.15). Relative to the LZDG, tGSH levels were significantly higher in the healthy control group (HG, 5.81 ± 0.10) and in all groups treated with single agents, including ATPG (5.83 ± 0.16), CQ10G (5.90 ± 0.28), PDXG (5.92 ± 0.27), and TPPG (5.94 ± 0.30), with all comparisons reaching statistical significance (*p* < 0.001).

Relative to the LZDG, administration of ATP (ATLG, 5.59 ± 0.14), CoQ10 (CQLG, 5.64 ± 0.12), pyridoxine (PXLG, 5.67 ± 0.11), and TPP (TPLG, 5.77 ± 0.05) effectively suppressed the linezolid-associated depletion of tGSH levels in sciatic nerve tissue (all *p* < 0.001). Intergroup comparisons demonstrated that tGSH levels did not differ significantly between the four co-treatment groups (all *p* > 0.05).

#### 2.3.3. Analysis of SOD Activity in Sciatic Nerve Tissue

**Figure 4:**
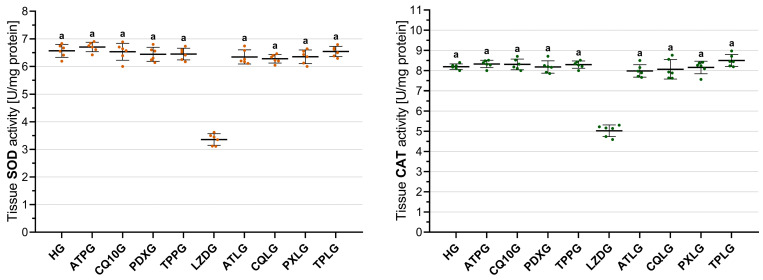
Effects of ATP, coenzyme Q10, pyridoxine, TPP, and linezolid on SOD and CAT activities in rat sciatic nerve tissue. Footnotes: All values are expressed as mean ± SD (standard deviation). All statistical analyses were performed using one-way ANOVA followed by Tukey’s honestly significant difference (HSD) post hoc tests. a indicates *p* < 0.001 vs. LZDG. For all groups, *n* = 6. Abbreviations: HG, healthy group; ATPG, ATP-only group; CQ10G, coenzyme Q10-only group; PDXG, pyridoxine-only group; TPPG, TPP-only group; LZDG, linezolid-only group; ATLG, ATP + linezolid; CQLG, coenzyme Q10 + linezolid; PXLG, pyridoxine + linezolid; TPLG, TPP + linezolid; ATP, adenosine triphosphate; TPP, thiamine pyrophosphate; SOD, superoxide dismutase; CAT, catalase.

As illustrated in Figure 4, sciatic nerve SOD activity was lowest in the linezolid-only group (LZDG, 3.36 ± 0.21). In comparison, SOD activity was significantly higher in the healthy control group (HG, 6.57 ± 0.24) and in all groups treated with single agents, including ATPG (6.70 ± 0.17), CQ10G (6.53 ± 0.31), PDXG (6.44 ± 0.25), and TPPG (6.45 ± 0.21), with all comparisons versus the LZDG reaching statistical significance (all *p* < 0.001).

Co-treatment with ATP (ATLG, 6.34 ± 0.26), CoQ10 (CQLG, 6.28 ± 0.15), pyridoxine (PXLG, 6.35 ± 0.25), and TPP (TPLG, 6.54 ± 0.19) significantly suppressed the linezolid-induced reduction in sciatic nerve SOD activity relative to the LZDG (all *p* < 0.001). SOD activity did not differ significantly between the four co-treatment groups upon intergroup comparison (all *p* > 0.05).

#### 2.3.4. Analysis of CAT Activity in Sciatic Nerve Tissue

Figure 4 shows that the sciatic nerve CAT activity was lowest in the linezolid-only group (LZDG, 5.03 ± 0.29). Compared with the LZDG, CAT activity was significantly higher in the healthy control group (HG, 8.19 ± 0.14) and in all groups treated with single agents, including ATPG (8.33 ± 0.19), CQ10G (8.31 ± 0.26), PDXG (8.18 ± 0.31), and TPPG (8.29 ± 0.18), with all comparisons versus the LZDG reaching statistical significance (all *p* < 0.001).

Co-treatment with ATP (ATLG, 7.98 ± 0.31), CoQ10 (CQLG, 8.06 ± 0.48), pyridoxine (PXLG, 8.15 ± 0.31), and TPP (TPLG, 8.50 ± 0.30) significantly suppressed the linezolid-induced reduction in sciatic nerve CAT activity compared with the LZDG (all *p* < 0.001). CAT activity exhibited comparable profiles between all four co-treatment groups, with no statistically significant differences detected (all *p* > 0.05).

#### 2.3.5. Analysis of Serum LDH Activity

**Figure 5:**
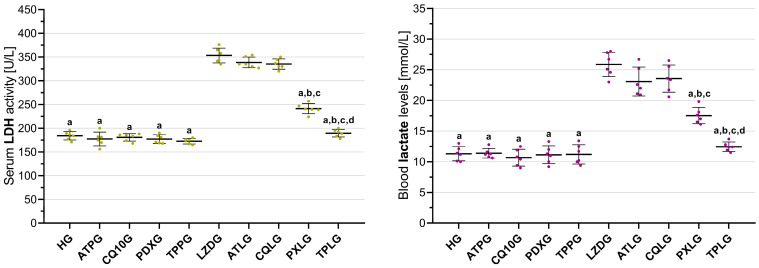
Effects of ATP, coenzyme Q10, pyridoxine, TPP, and linezolid on serum LDH activity and blood lactate levels in rats. Footnotes: All values are expressed as mean ± SD (standard deviation). All statistical analyses were performed using one-way ANOVA followed by Tukey’s honestly significant difference (HSD) post hoc test. Only statistically significant comparisons are indicated. a indicates *p* < 0.001 vs. LZDG, b indicates *p* < 0.001 vs. ATLG, c indicates *p* < 0.001 vs. CQLG, and d indicates *p* < 0.001 vs. PXLG. For all groups, *n* = 6. Abbreviations: HG, healthy group; ATPG, ATP-only group; CQ10G, coenzyme Q10-only group; PDXG, pyridoxine-only group; TPPG, TPP-only group; LZDG, linezolid-only group; ATLG, ATP + linezolid; CQLG, coenzyme Q10 + linezolid; PXLG, pyridoxine + linezolid; TPLG, TPP + linezolid; ATP, adenosine triphosphate; TPP, thiamine pyrophosphate; LDH, lactate dehydrogenase.

Figure 5 shows that the serum LDH activity was significantly elevated in the linezolid-only group (LZDG, 353.33 ± 15.64) compared with the healthy control group (HG, 184.00 ± 8.90) and all agent-only groups, including ATPG (177.33 ± 14.58), CQ10G (180.67 ± 7.89), PDXG (177.17 ± 9.11), and TPPG (172.67 ± 5.68), with all comparisons reaching statistical significance (LZDG vs. HG, ATPG, CQ10G, PDXG, and TPPG; all *p* < 0.001).

However, co-administration of ATP (ATLG, 338.67 ± 11.18) or CoQ10 (CQLG, 335.33 ± 10.98) did not result in a statistically significant reduction in serum LDH activity relative to the linezolid-only group (LZDG, 353.33 ± 15.64; *p* = 0.360 and *p* = 0.127, respectively). By comparison, co-treatment with pyridoxine (PXLG, 241.33 ± 10.82) and TPP (TPLG, 189.50 ± 8.02) significantly attenuated the linezolid-induced elevation in LDH activity (both *p* < 0.001).

Pairwise analyses further revealed no statistically significant difference in LDH activity between the ATP and CoQ10 co-treatment groups (*p* = 1.000). In turn, both the pyridoxine- and TPP-treated groups exhibited significantly lower LDH activity than either ATP or CoQ10 co-treatment (all *p* < 0.001). Notably, LDH activity was significantly lower in the TPP-treated group than in the pyridoxine-treated group (*p* < 0.001).

#### 2.3.6. Analysis of Blood Lactate Levels

Figure 5 illustrates a significant increase in blood lactate levels in the linezolid-only group (LZDG, 25.87 ± 1.97) relative to the healthy control group (HG, 11.30 ± 1.16) and all agent-only treatment groups, including ATPG (11.38 ± 0.78), CQ10G (10.67 ± 1.37), PDXG (11.13 ± 1.45), and TPPG (11.20 ± 1.58). These differences were statistically significant for all comparisons involving the LZDG (all *p* < 0.001).

Evaluation of combination treatments demonstrated that co-administration of ATP (ATLG, 23.08 ± 2.35) or CoQ10 (CQLG, 23.55 ± 2.22) failed to elicit a significant reduction in blood lactate levels compared with the linezolid-only group (*p* = 0.096 and *p* = 0.277, respectively). In contrast, animals receiving pyridoxine (PXLG, 17.52 ± 1.33) or TPP (TPLG, 12.45 ± 0.76) in combination with linezolid exhibited a significant decrease in blood lactate levels, with both interventions reaching statistical significance (*p* < 0.001).

Subsequent pairwise comparisons indicated no significant difference between the ATP and CoQ10 co-treatment groups (*p* = 1.000). Conversely, blood lactate levels in both the pyridoxine- and TPP-treated groups were significantly lower than those observed in the ATP or CoQ10 co-treatment groups (all *p* < 0.001). Moreover, blood lactate levels were significantly reduced in the TPP-treated group relative to the pyridoxine-treated group (*p* < 0.001).

## 3. Discussion

In this study, the potential protective and therapeutic effects of ATP, CoQ10, pyridoxine, and TPP in linezolid-induced peripheral neuropathic pain in rats were systematically investigated. In addition to their effects on linezolid-associated mechanical paw withdrawal thresholds, oxidant and antioxidant parameters, serum LDH activity, and lactate levels were also evaluated. Peripheral neuropathy is a well-recognized adverse effect of linezolid [27], with oxidative stress and mitochondrial dysfunction being considered fundamental mechanisms underlying drug-induced peripheral neuropathy [12]. In our study, the linezolid-treated group exhibited a significant decrease in paw withdrawal thresholds. Linezolid, particularly when administered at high doses and for prolonged durations, has been reported to induce peripheral neuropathy accompanied by increased sensory hypersensitivity [28,29]. Accordingly, the reduction in paw withdrawal thresholds observed in the linezolid-treated group may be attributable to the drug’s potential neurotoxic effects.

Adenosine triphosphate (ATP), investigated for its effects against linezolid-induced peripheral neuropathic pain, is synthesized in mitochondria through oxidative phosphorylation. The respiratory chain and ATP synthesis have been increasingly recognized as potential therapeutic targets for peripheral neuropathy [15]. The literature highlights that ATP exerts analgesic (antinociceptive) effects via activation of the A1 receptor [30]. In the group treated with ATP alone, paw withdrawal thresholds were observed to be close to those of the healthy control group. This finding indicates that ATP administration alone does not alter paw withdrawal thresholds in healthy tissues. However, the absence of significant ATP-mediated modulation of the LZD-induced reduction in paw withdrawal thresholds suggests that under the applied dosing regimen and experimental conditions, the antinociceptive efficacy of ATP may have been weaker than anticipated or potentially masked. Similarly, no significant change in paw withdrawal thresholds was observed in animals administered CoQ10 alone, nor did CoQ10 administration result in a significant improvement in the LZD-induced reduction in paw withdrawal thresholds. Our experimental findings indicate that the effects of CoQ10 do not fully align with previous reports demonstrating its antioxidant, anti-inflammatory [17], antinociceptive [31,32], and paw withdrawal threshold-enhancing [33] properties. In contrast, the results derived from the pyridoxine- and TPP-treated groups are in good agreement with previous reports. As is well-established, pyridoxine plays a critical role in axonal transport, and its deficiency may lead to the development of peripheral neuropathy [20,21]. The literature reports that combination therapies containing pyridoxine are associated with reduced peripheral neuropathy-related symptoms [20], and that pyridoxine attenuates pain perception in rat models [34]. In our study, pyridoxine significantly attenuated the linezolid-induced reduction in paw withdrawal thresholds; however, its protective efficacy was weaker than that observed with TPP. Thiamine pyrophosphate (TPP) is essential for energy metabolism, as the conversion of pyruvate to acetyl-CoA is catalyzed by the pyruvate dehydrogenase (PDH) complex in a TPP-dependent manner [35]. In the setting of TPP deficiency or inhibition of the PDH complex, pyruvate is preferentially shunted toward lactate formation via LDH [36]. As a result of the diversion of pyruvate toward lactate, lactic acid levels increase. This increase amplifies peripheral inflammation by promoting proinflammatory mediators, thereby enhancing pain sensitivity [37]. In our study, TPP emerged as the most effective agent in preventing the linezolid-induced reduction in paw withdrawal thresholds. This result is consistent with the findings of Rahman et al., which indicate that neuropathic pain is fundamentally associated with disturbances in energy metabolism [38]. Moreover, Onk et al. reported that TPP exerts protective effects on neural tissue through its antioxidant and anti-inflammatory properties [26], while Nisar et al. demonstrated that thiamine deficiency and elevated lactate levels are associated with neuropathic pain [39]. Collectively, these findings indicate that the metabolic and antioxidant properties of TPP may significantly ameliorate linezolid-induced pain hypersensitivity.

To assess the role of oxidative stress in pain-related mechanisms, various biomarkers were analyzed in the present study. Oxidative stress induces lipid peroxidation (LPO) of cellular fatty acids, and MDA, which is generated as a consequence of this process, is one of the most widely used biomarkers of LPO [40]. The observed increase in MDA levels in the linezolid-only group represents an oxidative stress response consistent with the literature documenting the mitochondrial toxicity of linezolid [41,42]. Our previous studies demonstrated that ATP attenuates the elevation in MDA levels associated with linezolid-induced oxidative damage [43], and that TPP reduces oxidative stress markers, leading to improved MDA levels [44]. Additionally, CoQ10 has been shown to suppress MDA increases in neurotoxicity models [45], and pyridoxine inhibits linezolid-induced oxidative stress and MDA elevation [46]. Collectively, these findings are consistent with our results, demonstrating that ATP, CoQ10, pyridoxine, and TPP comparably suppressed the linezolid-induced increase in MDA levels.

Glutathione (GSH), a key antioxidant parameter, scavenges ROS and protects cells against oxidative damage [47]. Linezolid is known to induce oxidative damage in hepatic tissue, leading to a reduction in tGSH levels [41]. The literature demonstrates that ATP prevents oxidative damage through modulation of tGSH levels [43], whereas CoQ10 suppresses oxidative-stress-induced depletion of tGSH [48]. Previous studies have reported that pyridoxine inhibits the decline in tGSH levels [46], while TPP restores tGSH levels, one of the key antioxidant parameters, toward normal values [49]. Our data demonstrate that the linezolid-induced reduction in tGSH levels was significantly prevented across all treatment groups. As is well-established, oxidative stress leads to a reduction in the activities of antioxidant enzymes such as SOD and CAT. Kendir-Demirkol et al. similarly reported a reduction in antioxidant enzyme activities under conditions of linezolid-induced oxidative damage [46], and our findings further corroborate these observations. Ozbay et al. demonstrated that ATP and CoQ10 suppress the decrease in SOD and CAT activities associated with drug-induced oxidative injury [50]. It has been reported that the linezolid-induced reductions in SOD and CAT activities are ameliorated by pyridoxine administration [46]. In addition, Isik et al. demonstrated that TPP inhibits the decrease in SOD and CAT activities associated with linezolid-induced oxidative damage [35]. Our results indicate that the decrease in SOD and CAT activities associated with linezolid administration was significantly inhibited in all treatment groups.

Lactate dehydrogenase (LDH), one of the control biomarkers assessed in our study, plays a critical role in anaerobic energy metabolism by catalyzing the conversion of pyruvate to lactate [36]. In contrast, the transport of pyruvate into mitochondria and its subsequent conversion to acetyl-CoA are mediated by the PDH enzyme complex. Thiamine pyrophosphate (TPP) serves as a critical cofactor required for the activation and optimal function of the PDH enzyme complex [35]. Under conditions of reduced PDH activity or TPP deficiency, pyruvate cannot enter the mitochondria and is instead diverted toward lactate production via LDH, resulting in increased LDH activity [51]. The diversion of pyruvate toward lactate results in increased lactic acid levels; this elevation promotes the release of proinflammatory mediators, thereby exacerbating peripheral inflammation and enhancing pain sensitivity [37]. The report by Chabrol et al. describing peripheral neuropathy in a patient with Leigh syndrome due to PDH deficiency demonstrates that the lack of PDH can disrupt energy metabolism, leading to lactic acid accumulation and consequent neurological manifestations [52]. While direct evidence linking linezolid to increased LDH activity is lacking, available data suggest that linezolid promotes lactate accumulation as a result of mitochondrial toxicity. This finding indicates an indirect metabolic shift in LDH-associated pyruvate–lactate metabolism. Our analyses reveal that LDH and lactate levels were highest in the linezolid-treated group, a finding that is consistent with the existing literature reporting linezolid-associated lactate accumulation and lactic acidosis [53,54]. Previous studies have reported that ATP exerts, albeit indirectly, a mitigating effect on linezolid-induced increases in LDH activity and lactate levels [55]. In contrast, ATP administration did not result in statistically significant suppression of the linezolid-induced elevations in LDH activity and lactate levels. The literature reports that CoQ10 reduces LDH levels [56], and some studies have also demonstrated its ability to attenuate lactate accumulation following intense physical exercise [57]. In contrast to ATP and CoQ10, pyridoxine significantly prevented the linezolid-induced increases in LDH activity and lactate levels. This finding is consistent with the study by Stockler et al., which reported lactic acidosis associated with pyridoxine deficiency [58]. Thiamine pyrophosphate (TPP) was the most effective agent in preventing the linezolid-induced increases in LDH activity and lactate levels. This finding is consistent with previous reports. It has been reported that TPP supplementation enhances aerobic performance in athletes by suppressing lactate levels [59], and in a previous study conducted with our colleagues, TPP administration was also shown to ameliorate elevated serum LDH activity and blood lactate levels in linezolid-induced hepatic injury [35]. Moreover, similar protective effects of TPP were reported in an amiodarone-induced neuropathy and neuropathic pain model in rats [60].

### Limitations

Several limitations of the present study should be acknowledged. First, the experimental design evaluated linezolid and the tested interventions at a single fixed dose and over a defined treatment duration; therefore, dose–response relationships and the temporal dynamics of neuropathy progression and recovery could not be assessed. Future studies employing multiple dosing regimens, extended follow-up periods, and combination treatment protocols involving different dosing ratios or multi-agent regimens are warranted to better characterize therapeutic windows and reversibility. Second, although the rat model reproduces key behavioral, oxidative, and metabolic features of linezolid-induced peripheral neuropathy observed in humans, species-specific differences in drug metabolism, nerve regeneration capacity, and pain processing may limit the direct extrapolation of these findings to clinical settings. Third, tissue or circulating concentrations of ATP, CoQ10, pyridoxine, and TPP were not measured. Accordingly, mechanistic interpretations regarding TPP’s effects are speculative and should be considered limited; the lack of pharmacokinetic data limits interpretation of exposure–response relationships and the degree to which mitochondrial cofactor availability was restored within peripheral nerve tissue. Fourth, while neuropathic pain was assessed using a validated mechanical paw withdrawal paradigm under blinded conditions, reliance on a single behavioral modality may restrict comprehensive phenotypic characterization, and incorporation of complementary assays evaluating tactile allodynia, thermal hyperalgesia, or cold sensitivity would strengthen behavioral profiling. Moreover, motor impairment or sedative effects were not formally assessed and may represent potential confounding factors. Additionally, the discrepancy between oxidative stress improvement and the lack of nociceptive benefit suggests that oxidative stress modulation alone may not be sufficient to relieve neuropathic pain. Fifth, direct electrophysiological assessments of peripheral nerve function, such as nerve conduction studies or electromyography, were not performed, which may limit objective functional confirmation. Sixth, histopathological, ultrastructural, and detailed molecular analyses of peripheral nerve and mitochondrial integrity were not conducted, thereby precluding direct evaluation of axonal degeneration, myelin pathology, and mitochondrial respiratory chain dysfunction. Seventh, despite the use of standardized and blinded experimental procedures, inherent biological variability and assay-related measurement variability may have contributed to minor variability in the observed outcomes. In addition, the small sample size (*n* = 6 per group) limits statistical power for multi-group comparisons and increases vulnerability to Type I and II errors. Although effect sizes were reported, formal adjustment for multiple comparisons was not systematically applied; therefore, the results should be interpreted cautiously and require confirmation in larger, adequately powered studies with pre-specified primary endpoints. Moreover, although several outcomes reached statistical significance, their biological and functional relevance may be limited, and statistical significance should not be interpreted as direct evidence of clinical effect. Eighth, only male rats were included in the present study; therefore, given documented sex-related differences in pain perception, oxidative stress responses, mitochondrial function, and peripheral nerve injury and repair, the generalizability of the findings to female subjects may be limited and warrants further investigation. Finally, the present study primarily focused on short-term behavioral, biochemical, and metabolic outcomes and did not evaluate the long-term persistence, progression, or reversibility of linezolid-induced neuropathic alterations. Consequently, the durability of the observed protective effects and their relevance to chronic exposure scenarios remain to be determined. Future studies incorporating longitudinal designs with extended observation periods will be essential to clarify the long-term trajectory of neuropathic changes and to further enhance the translational relevance of these findings.

## 4. Materials and Methods

### 4.1. Animals

In this experimental study, a total of 60 male albino Wistar rats aged 9–10 weeks with body weights ranging from 280 to 295 g were used. All animals were obtained from the Experimental Animals Application and Research Center of Erzincan Binali Yıldırım University (Erzincan, Turkey). The rats were randomly assigned to ten experimental groups (*n* = 6 per group) to ensure comparable mean body weights. Prior to experimental procedures, all animals underwent a 7-day acclimatization period and were housed in standard wire laboratory cages measuring 20 cm in height, 35 cm in width, and 55 cm in length, corresponding to a floor area of 1925 cm^2^, with six rats per cage. Throughout the study, animals were maintained under controlled environmental conditions, including a 12 h light/12 h dark photoperiod, a constant ambient temperature of 22 ± 2 °C, and relative humidity maintained between 30% and 70%. Standard laboratory chow (Bayramoglu Feed and Flour Industry Inc., Erzurum, Turkey) and tap water were provided ad libitum throughout the experimental period. All animals were clinically healthy and free from overt signs of disease at the start of the experiments.

All animal-related experimental procedures were conducted at the Experimental Animals Application and Research Center of Erzincan Binali Yıldırım University. The study was designed and performed in accordance with the European Parliament Directive 2010/63/EU on the protection of animals used for scientific purposes (Approval ID: 2016-24-199) and was carried out in compliance with the ARRIVE (Animal Research: Reporting of In Vivo Experiments) guidelines [61]. All efforts were made to minimize animal suffering and to reduce the number of animals used. The experimental design was reviewed and approved by the Local Animal Ethics Committee of Erzincan Binali Yıldırım University (Erzincan, Turkey; Approval No.: 46; Session: September 2025; Date: 25 September 2025).

### 4.2. Reagents and Chemicals

All reagents and chemical compounds used in the present study were of analytical grade and were sourced from commercial suppliers. Thiopental sodium (Pental Sodium^®^, 0.5 g powder for solution for injection; Catalog No.: 8699508270385) was obtained from Menarini Health and Pharmaceutical Industry Trade Inc. (Istanbul, Turkey). Linezolid (Zyvoxid^®^ 600 mg tablet; Catalog No.: 8681308091031) was purchased from Pfizer PFE Pharmaceuticals Inc. (Istanbul, Turkey). ATP sodium (Sodium Adenosine Triphosphate-Darnytsia^®^ 10 mg/mL injectable solution; Catalog No.: 4823006406180) was obtained from Darnytsia Pharmaceutical Co. (Kyiv, Ukraine). CoQ10 (CoQ-10^®^ 30 mg tablet; Catalog No.: 0033984009325) was supplied by Solgar (Leonia, NJ, USA). Pyridoxine hydrochloride (B6Vigen^®^ 50 mg tablet; Catalog No.: 8699774010050) was obtained from Aksu Pharma Medical Products Pharmaceutical Industry and Trade Ltd. (Istanbul, Turkey). TPP (cocarboxylase hydrochloride^®^; 50 mg/2 mL injectable formulation; Catalog No.: 4820011070436) was supplied by BioPharma (Kyiv, Ukraine).

### 4.3. Experimental Design and Randomization

The sample size was determined in accordance with the 4R guidelines (Reduction, Refinement, Replacement, and Responsibility), adhering to the principle of employing the minimum number of animals required to generate robust and reproducible findings [62]. Exclusion criteria were prospectively defined for two distinct phases of the experimental protocol (pre-experimental and peri/post-experimental).

Pre-experimental exclusion criteria comprised abnormal body posture, reduced spontaneous activity, or injuries resulting from aggressive interactions between cage mates; animals meeting these criteria were excluded prior to randomization and initiation of the experimental interventions. Peri-experimental and post-experimental exclusion criteria included unexpected mortality before the planned endpoints or complications related to anesthesia or drug administration; dosing-related errors such as unsuccessful oral gavage or extravasation during injection; deviations from the predefined treatment schedule or incomplete administration of the study compounds; body weight loss exceeding 15–20% of baseline values, signs of dehydration, or manifestations of systemic illness; severe distress indicative of uncontrolled pain or suffering, including self-injurious behavior or persistent vocalization; the inability to complete behavioral assessments due to incompatibility unrelated to the intervention or motor impairments unrelated to the experimental interventions; and a loss of tissue integrity during collection or processing that compromised the reliability of biochemical analyses.

These exclusion criteria were applied systematically throughout the intervention period and during subsequent data evaluation. No animals met the predefined exclusion criteria at any stage of the study, and consequently, no subjects were excluded from the final analyses. Group allocation was performed using a random number table to ensure unbiased assignment. To further minimize potential confounding effects and systematic sources of bias, each cage and each individual animal was labeled with a numerical code that was consistently maintained throughout the entire experimental period. Behavioral assessments and biochemical analyses were performed by investigators blinded to group allocation.

### 4.4. Experimental Groups

Following random allocation, the animals were assigned to ten experimental groups. These included a healthy control group (HG), an ATP-only group (ATPG), a CoQ10-only group (CQ10G), a pyridoxine-only group (PDXG), a TPP-only group (TPPG), a linezolid-only group (LZDG), an ATP plus linezolid group (ATLG), a CoQ10 plus linezolid group (CQLG), a pyridoxine plus linezolid group (PXLG), and a TPP plus linezolid group (TPLG).

### 4.5. Experimental Procedure

Initially, baseline mechanical paw withdrawal thresholds were assessed in all groups using a Basile analgesimeter based on the Randall–Selitto method [63]. The Randall–Selitto assay constitutes a quantitative nociceptive assessment method in which graded, linearly increasing mechanical pressure is applied to the dorsal surface of the rat hind paw to elicit and measure withdrawal responses [64]. Animals assigned to the ATPG (*n* = 6) and ATLG (*n* = 6) groups received ATP at a dose of 5 mg/kg, those in the CQ10G (*n* = 6) and CQLG (*n* = 6) groups received CoQ10 at a dose of 10 mg/kg, animals in the PDXG (*n* = 6) and PXLG (*n* = 6) groups received pyridoxine at a dose of 50 mg/kg, and animals in the TPPG (*n* = 6) and TPLG (*n* = 6) groups received TPP at a dose of 20 mg/kg. Adenosine triphosphate (ATP) and TPP were administered intraperitoneally once daily, whereas CoQ10 was administered once daily and pyridoxine twice daily by oral gavage. The doses and routes of administration of ATP [60], CoQ10 [50], pyridoxine [65], and TPP [60] were selected based on previously validated experimental models demonstrating consistent antioxidant and tissue-protective effects. Animals in the healthy control group (HG, *n* = 6) and the linezolid-treated group (LZDG, *n* = 6) received distilled water orally and served as vehicle controls.

One hour after the administration of distilled water or the respective treatments, animals in LZDG, ATLG, CQLG, PXLG, and TPLG were administered linezolid at a dose of 125 mg/kg by oral gavage twice daily at 12 h intervals. This dosing protocol was derived from established rat models demonstrating that oral administration of linezolid at 125 mg/kg twice daily reproducibly induces neuropathy [43]. This dosing regimen was maintained for 14 consecutive days. Mechanical paw withdrawal thresholds were determined in all experimental groups immediately prior to the initiation of the protocol and re-evaluated after 14 days of treatment, following the same standardized procedure. At each assessment point, three consecutive measurements were obtained per animal and averaged for statistical analysis; terminal evaluations were conducted 1 h after the final administered dose in the single-agent and treatment groups, whereas the healthy control group was assessed concurrently under identical experimental conditions.

At the conclusion of the experimental period, rats were euthanized under deep anesthesia induced by thiopental sodium administered at a dose of 50 mg/kg. Subsequently, sciatic nerve tissues were carefully harvested. Levels of malondialdehyde (MDA), total glutathione (tGSH), superoxide dismutase (SOD), and catalase (CAT) were quantified in the excised sciatic nerve tissues. Prior to euthanasia, blood samples were collected from the tail vein to determine lactate dehydrogenase (LDH) activity and lactate concentrations. All experimental data were subsequently analyzed and compared between the study groups.

### 4.6. Biochemical Analyses

#### 4.6.1. Preparation of Samples

Sciatic nerve specimens were meticulously dissected from each animal and immediately rinsed in ice-cold 0.9% sodium chloride solution to remove residual blood and extraneous tissue contaminants. Approximately 20–30 mg of sciatic nerve tissue per animal was accurately weighed, finely sectioned, and rapidly cryopreserved by immersion in liquid nitrogen. The frozen tissue fragments were subsequently ground into a homogeneous powder using a pre-chilled mortar and pestle. The resulting tissue powder was homogenized in phosphate-buffered saline (PBS, pH 7.4) at a 1:10 (*w*/*v*) ratio. Homogenates were briefly vortex-mixed for 10 s and then centrifuged at 10,000× *g* for 20 min at 4 °C. Following centrifugation, the supernatant fraction was carefully isolated and preserved at −80 °C until further biochemical evaluation. To ensure analytical consistency and enable reliable intergroup comparisons, all biochemical measurements were normalized to total protein content and expressed as nmol/mg protein for MDA and tGSH, and as U/mg protein for SOD and CAT activities.

#### 4.6.2. Determination of MDA, Total GSH, SOD, CAT, and Protein in Sciatic Nerve Tissue

Malondialdehyde (MDA) and tGSH levels and SOD and CAT activities in sciatic nerve tissue were measured using rat-specific ELISA kits (Catalog No.: ELK8612 for MDA; ELK8577 for tGSH; ELK8178 for SOD; ELK5986 for CAT; ELK Biotechnology^®^ Co., Ltd., Sugar Land, TX, USA) in accordance with the manufacturer’s instructions. Total protein content was determined using a Bradford-based colorimetric assay [66].

#### 4.6.3. Determination of Serum LDH Activity

Quantitative measurement of serum LDH (P–L) activity was performed spectrophotometrically using a Beckman Coulter AU5800 automated analyzer (Beckman Coulter Inc.^®^, Brea, CA, USA, 2023).

#### 4.6.4. Determination of Blood Lactate Levels

Blood samples were obtained from the tail vein of rats using lithium heparin-coated, pre-heparinized syringes to inhibit coagulation and limit glycolytic activity. Blood lactate levels were subsequently determined with an ABL800 FLEX blood gas analyzer (Radiometer^®^, Copenhagen, Denmark) following the manufacturer’s protocol.

### 4.7. Statistical Analyses

All statistical analyses of biochemical parameters and mechanical paw withdrawal thresholds were performed using IBM SPSS^®^ Statistics for Windows (Version 27.0; IBM Corp., Armonk, NY, USA, 2020). Graphical data visualizations were prepared using GraphPad Prism^®^ (Version 8.0.1; GraphPad Software, San Diego, CA, USA, 2018). Data are presented as the mean ± standard deviation (SD). The normality of the data distribution for the biochemical parameters was evaluated using the Shapiro–Wilk test (Table S1), whereas the assumption of homogeneity of variances across groups was assessed using Levene’s test (Table S2). When both assumptions were satisfied, between-group comparisons were conducted using one-way analysis of variance (ANOVA), followed by Tukey’s honestly significant difference (HSD) test for post hoc pairwise comparisons. In cases where the homogeneity of variances assumption was violated, Welch’s ANOVA was applied, and multiple comparisons were subsequently performed using the Games–Howell test (Table S3). Statistical analyses of mechanical paw withdrawal thresholds were performed using Δ (post–pre) values. Normality was assessed with the Shapiro–Wilk test, and within-group pre–post comparisons were conducted using two-tailed paired-samples *t*-tests (Table S4). For between-group analyses, normality and homogeneity of variances were evaluated using the Shapiro–Wilk and Levene’s tests, respectively; group comparisons were performed using Welch’s ANOVA followed by Games–Howell post hoc multiple comparisons (Tables S5 and S6). Statistical significance was defined as a *p* value < 0.05.

## 5. Conclusions

This study demonstrated that linezolid induces peripheral neuropathic pain in rats, characterized by reduced mechanical paw withdrawal thresholds, increased oxidative stress, and impaired energy metabolism. While ATP and CoQ10 preserved oxidative stress parameters, they did not significantly improve paw withdrawal thresholds. In contrast, pyridoxine and particularly TPP produced marked improvements in behavioral thresholds and associated biochemical indices.

The ability of TPP to most effectively preserve paw withdrawal thresholds and suppress increases in LDH and lactate levels highlights the importance of pyruvate–lactate balance and energy metabolism in the pathogenesis of neuropathic pain. These findings indicate that TPP may be the most effective protective agent against linezolid-induced peripheral neuropathic pain and underscore the potential relevance of metabolically targeted strategies for preventing neuropathic complications; however, mechanistic interpretations regarding TPP’s effects remain speculative and should be considered limited.

## Data Availability

The original contributions presented in this study are included in the article/Supplementary Materials. Further inquiries can be directed to the corresponding author.

## Supplementary Materials

The following supporting information can be downloaded at https://www.mdpi.com/article/10.3390/ph19020341/s1, Table S1: Shapiro–Wilk test-based assessment of normality for sciatic nerve tissue and blood biochemical parameters in rats; Table S2: Assessment of variance homogeneity for biochemical variables using Levene’s test; Table S3: Comparison of *p*-values for oxidant and antioxidant markers in rat sciatic nerve tissue and blood LDH and lactate; Table S4: Within-group analysis of Δ (post–pre) mechanical paw withdrawal thresholds following treatment; Table S5: Between-group analysis of Δ (post–pre) mechanical paw withdrawal thresholds and corresponding relative restoration of mechanical threshold; Table S6: Between-group comparison of pre-, post-treatment and Δ (Post–Pre) mechanical paw withdrawal thresholds.
