# Supplementary material for: A Comparative Evaluation of the Therapeutic Effects of Adenosine Triphosphate, Coenzyme Q10, Pyridoxine, and Thiamine Pyrophosphate in a Linezolid-Induced Peripheral Neuropathic Pain Model in Rats
Source: Pharmaceuticals (Basel). 2026 Feb 22;19(2):341. doi: 10.3390/ph19020341 (PMC12944494; doi:10.3390/ph19020341)
Supplement: Supplementary file 1 [file pharmaceuticals-19-00341-s001.zip › Table S1-R2.pdf]

Table S1. Shapiro–Wilk test-based assessment of normality for sciatic nerve tissue and blood biochemical parameters in rats.

			Biochemical Variables					
		**Shapiro-Wilk**	**MDA**	**tGSH**	**SOD**	**CAT**	**LDH**	**Lactate**
Groups	HG	Statistic	0.924	0.922	0.957	0.988	0.981	0.942
		df	6	6	6	6	6	6
		Sig.	0.535	0.516	0.799	0.984	0.955	0.672
	ATPG	Statistic	0.988	0.975	0.960	0.906	0.974	0.891
		df	6	6	6	6	6	6
		Sig.	0.984	0.922	0.819	0.409	0.921	0.323
	CQ10G	Statistic	0.989	0.891	0.937	0.955	0.864	0.954
		df	6	6	6	6	6	6
		Sig.	0.988	0.326	0.634	0.783	0.205	0.774
	PDXG	Statistic	0.888	0.934	0.942	0.906	0.892	0.989
		df	6	6	6	6	6	6
		Sig.	0.309	0.613	0.676	0.408	0.330	0.986
	TPPG	Statistic	0.937	0.812	0.970	0.971	0.945	0.931
		df	6	6	6	6	6	6
		Sig.	0.636	0.075	0.895	0.901	0.700	0.588
	LZDG	Statistic	0.912	0.919	0.912	0.846	0.948	0.933
		df	6	6	6	6	6	6
		Sig.	0.452	0.499	0.453	0.147	0.724	0.606
	ATLG	Statistic	0.925	0.866	0.849	0.932	0.865	0.880
		df	6	6	6	6	6	6
		Sig.	0.545	0.211	0.154	0.593	0.206	0.267
	CQLG	Statistic	0.858	0.944	0.962	0.854	0.964	0.965
		df	6	6	6	6	6	6
		Sig.	0.181	0.693	0.833	0.169	0.851	0.858
	PXLG	Statistic	0.939	0.991	0.951	0.811	0.948	0.936
		df	6	6	6	6	6	6
		Sig.	0.648	0.991	0.746	0.073	0.721	0.627
	TPLG	Statistic	0.958	0.959	0.975	0.922	0.983	0.960
		df	6	6	6	6	6	6
		Sig.	0.806	0.816	0.923	0.523	0.966	0.820

Footnotes: The distributions of MDA, tGSH, SOD, and CAT in sciatic nerve tissue and LDH and lactate in blood were consistent with the assumption of normality according to the Shapiro–Wilk test; therefore, group comparisons were performed using ANOVA. For all groups, $n = 6$.

Abbreviations: HG, healthy group; ATPG, ATP-alone group; CQ10G, coenzyme Q10-alone group; PDXG, pyridoxine-alone group; TPPG, TPP-alone group; LZDG, linezolid-alone group; ATLG, ATP + linezolid; CQLG, coenzyme Q10 + linezolid; PXLG, pyridoxine + linezolid; TPLG, TPP + linezolid; ATP, adenosine triphosphate; TPP, thiamine pyrophosphate; MDA, malondialdehyde; tGSH, total glutathione; SOD, superoxide dismutase; CAT, catalase; LDH, lactate dehydrogenase; df, degrees of freedom; Sig, significance.
