# Supplementary material for: A Comparative Evaluation of the Therapeutic Effects of Adenosine Triphosphate, Coenzyme Q10, Pyridoxine, and Thiamine Pyrophosphate in a Linezolid-Induced Peripheral Neuropathic Pain Model in Rats
Source: Pharmaceuticals (Basel). 2026 Feb 22;19(2):341. doi: 10.3390/ph19020341 (PMC12944494; doi:10.3390/ph19020341)
Supplement: Supplementary file 1 [file pharmaceuticals-19-00341-s001.zip › Table S2-R2.pdf]

Table S2. Assessment of variance homogeneity for biochemical variables using Levene's test.

	Biochemical Variables					
	**MDA**	**tGSH**	**SOD**	**CAT**	**LDH**	**Lactate**
**Levene's statistic**	2.444	7.865	0.572	1.630	1.144	1.975
**df1**	9	9	9	9	9	9
**df2**	50	50	50	50	50	50
**Sig.**	0.022	<0.001	0.813	0.132	0.351	0.062

Footnotes: Following assessment of variance homogeneity, the Games–Howell test was used for MDA and tGSH due to violation of this assumption, whereas Tukey's Honestly Significant Difference (HSD) test was applied for post hoc comparisons of SOD, CAT, LDH, and lactate. For all groups, $n = 6$.
Abbreviations: MDA, malondialdehyde; tGSH, total glutathione; SOD, superoxide dismutase; CAT, catalase; LDH, lactate dehydrogenase; df, degrees of freedom; Sig, significance.
