# Supplementary material for: A Comparative Evaluation of the Therapeutic Effects of Adenosine Triphosphate, Coenzyme Q10, Pyridoxine, and Thiamine Pyrophosphate in a Linezolid-Induced Peripheral Neuropathic Pain Model in Rats
Source: Pharmaceuticals (Basel). 2026 Feb 22;19(2):341. doi: 10.3390/ph19020341 (PMC12944494; doi:10.3390/ph19020341)
Supplement: Supplementary file 1 [file pharmaceuticals-19-00341-s001.zip › Table S3-R2.pdf]

Table S3. Comparison of p-values for oxidant and antioxidant markers in rat sciatic nerve tissue and blood LDH and lactate.

	Post hoc *p*-values					
**Group comparisons**	**MDA***	**tGSH***	**SOD****	**CAT****	**LDH****	**Lactate****
HG vs. ATPG	1.000	1.000	0.987	0.998	0.984	1.000
HG vs. CQ10G	0.996	0.998	1.000	1.000	1.000	0.999
HG vs. PDXG	0.977	0.988	0.994	1.000	0.982	1.000
HG vs. TPPG	0.847	0.980	0.996	1.000	0.709	1.000
HG vs. LZDG	<0.001	<0.001	<0.001	<0.001	<0.001	<0.001
HG vs. ATLG	0.928	0.154	0.796	0.958	<0.001	<0.001
HG vs. CQLG	0.855	0.284	0.471	0.999	<0.001	<0.001
HG vs. PXLG	0.912	0.449	0.816	1.000	<0.001	<0.001
HG vs. TPLG	0.999	0.980	1.000	0.717	0.996	0.958
ATPG vs. CQ10G	1.000	1.000	0.947	1.000	1.000	0.999
ATPG vs. PDXG	0.999	0.998	0.598	0.996	1.000	1.000
ATPG vs. TPPG	0.970	0.996	0.632	1.000	0.999	1.000
ATPG vs. LZDG	<0.001	<0.001	<0.001	<0.001	<0.001	<0.001
ATPG vs. ATLG	0.788	0.253	0.184	0.561	<0.001	<0.001
ATPG vs. CQLG	0.564	0.448	0.058	0.857	<0.001	<0.001
ATPG vs. PXLG	0.596	0.624	0.199	0.989	<0.001	<0.001
ATPG vs. TPLG	0.971	0.986	0.961	0.989	0.621	0.974
CQ10G vs. PDXG	1.000	1.000	0.999	0.999	1.000	1.000
CQ10G vs. TPPG	0.999	1.000	1.000	1.000	0.950	1.000
CQ10G vs. LZDG	<0.001	<0.001	<0.001	<0.001	<0.001	<0.001
CQ10G vs. ATLG	0.663	0.425	0.910	0.647	<0.001	<0.001
CQ10G vs. CQLG	0.381	0.594	0.640	0.909	<0.001	<0.001
CQ10G vs. PXLG	0.379	0.713	0.922	0.996	<0.001	<0.001
CQ10G vs. TPLG	0.878	0.959	1.000	0.976	0.911	0.637
PDXG vs. TPPG	1.000	1.000	1.000	0.999	0.999	1.000
PDXG vs. LZDG	<0.001	<0.001	<0.001	<0.001	<0.001	<0.001

PDXG vs. ATLG	0.587	0.313	0.999	0.972	<0.001	<0.001
PDXG vs. CQLG	0.350	0.463	0.961	0.999	<0.001	<0.001
PDXG vs. PXLG	0.375	0.578	0.999	1.000	<0.001	<0.001
PDXG vs. TPLG	0.800	0.890	0.999	0.666	0.603	0.909
TPPG vs. LZDG	<0.001	<0.001	<0.001	<0.001	<0.001	<0.001
TPPG vs. ATLG	0.438	0.348	0.998	0.692	<0.001	<0.001
TPPG vs. CQLG	0.124	0.491	0.950	0.931	<0.001	<0.001
TPPG vs. PXLG	0.059	0.598	0.999	0.998	<0.001	<0.001
TPPG vs. TPLG	0.505	0.882	0.999	0.966	0.190	0.932
LZDG vs. ATLG	<0.001	<0.001	<0.001	<0.001	0.360	0.096
LZDG vs. CQLG	<0.001	<0.001	<0.001	<0.001	0.127	0.277
LZDG vs. PXLG	<0.001	<0.001	<0.001	<0.001	<0.001	<0.001
LZDG vs. TPLG	<0.001	<0.001	<0.001	<0.001	<0.001	<0.001
ATLG vs. CQLG	1.000	0.999	1.000	1.000	1.000	1.000
ATLG vs. PXLG	1.000	0.958	1.000	0.989	<0.001	<0.001
ATLG vs. TPLG	0.996	0.258	0.886	0.087	<0.001	<0.001
CQLG vs. PXLG	1.000	1.000	1.000	1.000	<0.001	<0.001
CQLG vs. TPLG	0.995	0.450	0.598	0.250	<0.001	<0.001
PXLG vs. TPLG	1.000	0.675	0.901	0.568	<0.001	<0.001
F-value	184.170[a]	212.876[a]	114.113	73.843	309.840	89.659
df (df1 / df2)	9 / 20.270	9 / 19.935	9 / 50	9 / 50	9 / 50	9 / 50
p	<0.001[b]	<0.001[b]	<0.001	<0.001	<0.001	<0.001

Footnotes: * Welch's analysis of variance (ANOVA) was used for statistical analyses, with post hoc multiple comparisons performed using the Games–Howell test. ** All statistical analyses were performed using one-way ANOVA, followed by Tukey's Honestly Significant Difference (HSD) test for post hoc comparisons. a indicates asymptotically F-distributed statistics, and b indicates p-values obtained from Welch's ANOVA. For all groups, *n* = 6.

Abbreviations: HG, healthy group; ATPG, ATP-alone group; CQ10G, coenzyme Q10-alone group; PDXG, pyridoxine-alone group; TPPG, TPP-alone group; LZDG, linezolid-alone group; ATLG, ATP + linezolid; CQLG, coenzyme Q10 + linezolid; PXLG, pyridoxine + linezolid; TPLG, TPP + linezolid; ATP, adenosine triphosphate; TPP, thiamine pyrophosphate; MDA, malondialdehyde; tGSH, total glutathione; SOD, superoxide dismutase; CAT, catalase; LDH, lactate dehydrogenase; df, degrees of freedom.
