# Supplementary material for: A Comparative Evaluation of the Therapeutic Effects of Adenosine Triphosphate, Coenzyme Q10, Pyridoxine, and Thiamine Pyrophosphate in a Linezolid-Induced Peripheral Neuropathic Pain Model in Rats
Source: Pharmaceuticals (Basel). 2026 Feb 22;19(2):341. doi: 10.3390/ph19020341 (PMC12944494; doi:10.3390/ph19020341)
Supplement: Supplementary file 1 [file pharmaceuticals-19-00341-s001.zip › Table S4-R2.pdf]

Table S4. Within-group analysis of Δ (post–pre) mechanical paw withdrawal thresholds following treatment.

		Paw withdrawal thresholds (g)								
**Groups**	***n***	**Pre-treatment**	**Post-treatment**	**Δ (Post–Pre)**	**Shapiro–Wilk pre-treatment *p* values**	**Shapiro–Wilk post-treatment *p* values**	**Shapiro-Wilk Δ (Post–Pre) *p*-values**	**t**	***p* (two-tailed)**	**Cohen's dz**
HG	6	33.00 ± 4.43	31.00 ± 4.56	-2.00 ± 6.99	0.897	0.497	0.944	-0.701	0.514	-0.286
ATPG	6	31.00 ± 3.10	30.00 ± 3.74	-1.00 ± 5.25	0.856	0.961	0.290	-0.466	0.661	-0.190
CQ10G	6	35.00 ± 4.47	32.00 ± 3.16	-3.00 ± 4.86	0.150	0.949	0.230	-1.513	0.191	-0.618
PDXG	6	30.00 ± 2.83	29.00 ± 3.41	-1.00 ± 2.61	0.523	0.334	0.039	-0.939	0.391	-0.383
TPPG	6	32.00 ± 3.63	30.00 ± 4.34	-2.00 ± 6.32	0.210	0.670	0.565	-0.775	0.474	-0.316
LZDG	6	36.00 ± 4.43	11.00 ± 1.90	-25.00 ± 2.76	0.897	0.113	0.899	-22.213	<0.001	-9.068
ATLG	6	33.00 ± 3.41	13.00 ± 1.79	-20.00 ± 2.28	0.141	0.607	0.277	-21.483	<0.001	-8.771
CQLG	6	34.00 ± 2.61	15.00 ± 1.79	-19.00 ± 4.15	0.122	0.607	0.118	-11.222	<0.001	-4.581
PXLG	6	32.00 ± 2.37	17.00 ± 1.90	-15.00 ± 1.41	0.739	0.113	0.960	-25.981	<0.001	-10.607
TPLG	6	35.00 ± 1.90	32.00 ± 2.90	-3.00 ± 1.79	0.113	0.271	0.607	-4.108	0.009	-1.677

Footnotes: Values are expressed as mean ± SD (standard deviation). Δ indicates the change between post- and pre-treatment values (post − pre). Normality of Δ values was assessed using the Shapiro–Wilk test. Although a deviation from normality was observed in one group ($p = 0.039$), two-tailed paired-samples t-tests were applied, as they are robust to minor departures from normality, particularly in small samples. Effect sizes are reported as Cohen's dz. Statistical significance was set at $p < 0.05$. For all groups, $n = 6$.

Abbreviations: HG, healthy group; ATPG, ATP-alone group; CQ10G, coenzyme Q10-alone group; PDXG, pyridoxine-alone group; TPPG, TPP-alone group; LZDG, linezolid-alone group; ATLG, ATP + linezolid; CQLG, coenzyme Q10 + linezolid; PXLG, pyridoxine + linezolid; TPLG, TPP + linezolid; ATP, adenosine triphosphate; TPP, thiamine pyrophosphate.
