# Supplementary material for: A Comparative Evaluation of the Therapeutic Effects of Adenosine Triphosphate, Coenzyme Q10, Pyridoxine, and Thiamine Pyrophosphate in a Linezolid-Induced Peripheral Neuropathic Pain Model in Rats
Source: Pharmaceuticals (Basel). 2026 Feb 22;19(2):341. doi: 10.3390/ph19020341 (PMC12944494; doi:10.3390/ph19020341)
Supplement: Supplementary file 1 [file pharmaceuticals-19-00341-s001.zip › Table S5-R2.pdf]

Table S5. Between-group analysis of Δ (post–pre) mechanical paw withdrawal thresholds and corresponding relative restoration of mechanical threshold.

Groups	Paw withdrawal thresholds (g) Δ (Post–Pre)	Shapiro-Wilk Δ (Post–Pre) *p*-values	Relative restoration of mechanical threshold (%)
HG	-2.00 ± 6.99	0.944	92.00
ATPG	-1.00 ± 5.25	0.290	96.00
CQ10G	-3.00 ± 4.86	0.230	88.00
PDXG	-1.00 ± 2.61	0.039	96.00
TPPG	-2.00 ± 6.32	0.565	92.00
LZDG	-25.00 ± 2.76	0.899	-
ATLG	-20.00 ± 2.28	0.277	20.00
CQLG	-19.00 ± 4.15	0.118	24.00
PXLG	-15.00 ± 1.41	0.960	40.00
TPLG	-3.00 ± 1.79	0.607	88.00

Footnotes: Values are expressed as mean ± SD (standard deviation). Δ values represent the change in paw withdrawal thresholds (post–pre). Negative Δ values indicate a reduction in paw withdrawal thresholds following treatment. Normality of Δ values was assessed using the Shapiro–Wilk test. Although a deviation from normality was observed in one group ($p = 0.039$), and variance homogeneity was violated as indicated by Levene's test ($p = 0.004$), between-group comparisons were performed using Welch's ANOVA, which is robust to violations of normality and variance heterogeneity, followed by Games–Howell post hoc multiple comparisons. Relative restoration of mechanical threshold (%) was calculated relative to the linezolid-alone group (LZDG), which was considered the disease control, using the following formula: [(Δ LZDG – Δ Treatment) / Δ LZDG] × 100. For all groups, $n = 6$.
Abbreviations: HG, healthy group; ATPG, ATP-alone group; CQ10G, coenzyme Q10-alone group; PDXG, pyridoxine-alone group; TPPG, TPP-alone group; LZDG, linezolid-alone group; ATLG, ATP + linezolid; CQLG, coenzyme Q10 + linezolid; PXLG, pyridoxine + linezolid; TPLG, TPP + linezolid; ATP, adenosine triphosphate; TPP, thiamine pyrophosphate.
