# Supplementary material for: A Comparative Evaluation of the Therapeutic Effects of Adenosine Triphosphate, Coenzyme Q10, Pyridoxine, and Thiamine Pyrophosphate in a Linezolid-Induced Peripheral Neuropathic Pain Model in Rats
Source: Pharmaceuticals (Basel). 2026 Feb 22;19(2):341. doi: 10.3390/ph19020341 (PMC12944494; doi:10.3390/ph19020341)
Supplement: Supplementary file 1 [file pharmaceuticals-19-00341-s001.zip › Table S6-R2.pdf]

Table S6. Between-group comparison of pre-, post-treatment and Δ (Post–Pre) mechanical paw withdrawal thresholds.

	Post hoc *p*-values		
**Group comparisons**	**Pre-treatment***	**Post-treatment***	**Δ (Post–Pre)****
HG vs. ATPG	0.990	1.000	1.000
HG vs. CQ10G	0.990	1.000	1.000
HG vs. PDXG	0.880	0.981	1.000
HG vs. TPPG	1.000	1.000	1.000
HG vs. LZDG	0.880	<0.001	0.003
HG vs. ATLG	1.000	<0.001	0.014
HG vs. CQLG	1.000	<0.001	0.016
HG vs. PXLG	1.000	<0.001	0.066
HG vs. TPLG	0.990	1.000	1.000
ATPG vs. CQ10G	0.589	0.981	0.999
ATPG vs. PDXG	1.000	1.000	1.000
ATPG vs. TPPG	1.000	1.000	1.000
ATPG vs. LZDG	0.281	<0.001	<0.001
ATPG vs. ATLG	0.990	<0.001	0.002
ATPG vs. CQLG	0.880	<0.001	0.002
ATPG vs. PXLG	1.000	<0.001	0.013
ATPG vs. TPLG	0.589	0.981	0.991
CQ10G vs. PDXG	0.281	0.808	0.992
CQ10G vs. TPPG	0.880	0.981	1.000
CQ10G vs. LZDG	1.000	<0.001	<0.001
CQ10G vs. ATLG	0.990	<0.001	0.002
CQ10G vs. CQLG	1.000	<0.001	0.003
CQ10G vs. PXLG	0.880	<0.001	0.018
CQ10G vs. TPLG	1.000	1.000	1.000
PDXG vs. TPPG	0.990	1.000	1.000
PDXG vs. LZDG	0.099	<0.001	<0.001

PDXG vs. ATLG	0.880	<0.001	<0.001
PDXG vs. CQLG	0.589	<0.001	<0.001
PDXG vs. PXLG	0.990	<0.001	<0.001
PDXG vs. TPLG	0.281	0.808	0.843
TPPG vs. LZDG	0.589	<0.001	0.002
TPPG vs. ATLG	1.000	<0.001	0.008
TPPG vs. CQLG	0.990	<0.001	0.009
TPPG vs. PXLG	1.000	<0.001	0.043
TPPG vs. TPLG	0.880	0.981	1.000
LZDG vs. ATLG	0.880	0.981	0.110
LZDG vs. CQLG	0.990	0.456	0.220
LZDG vs. PXLG	0.589	0.047	0.001
LZDG vs. TPLG	1.000	<0.001	<0.001
ATLG vs. CQLG	1.000	0.981	1.000
ATLG vs. PXLG	1.000	0.456	0.029
ATLG vs. TPLG	0.990	<0.001	<0.001
CQLG vs. PXLG	0.990	0.981	0.515
CQLG vs. TPLG	1.000	<0.001	0.001
PXLG vs. TPLG	0.880	<0.001	<0.001
F-value	1.865	47.599	51.216[a]
df (df1 / df2)	9 / 50	9 / 50	9 / 20.120
p	0.079	<0.001	<0.001[b]

Footnotes: Δ values represent the change in paw withdrawal thresholds after and before treatment. *Statistical analyses were performed using one-way analysis of variance (ANOVA), followed by Tukey's Honestly Significant Difference (HSD) post hoc multiple comparisons. **Statistical analyses were performed using Welch's ANOVA, followed by Games–Howell post hoc multiple comparisons. Superscript a denotes asymptotically F-distributed statistics, whereas superscript b indicates p-values derived from Welch's ANOVA. For all groups, *n* = 6.
Abbreviations: HG, healthy group; ATPG, ATP-alone group; CQ10G, coenzyme Q10-alone group; PDXG, pyridoxine-alone group; TPPG, TPP-alone group; LZDG, linezolid-alone group; ATLG, ATP + linezolid; CQLG, coenzyme Q10 + linezolid; PXLG, pyridoxine + linezolid; TPLG, TPP + linezolid; ATP, adenosine triphosphate; TPP, thiamine pyrophosphate; df, degrees of freedom.
